# Cre-Dependent Anterograde Transsynaptic Labeling and Functional Imaging in Zebrafish Using VSV With Reduced Cytotoxicity

**DOI:** 10.3389/fnana.2021.758350

**Published:** 2021-10-06

**Authors:** Stanislav Kler, Manxiu Ma, Sujatha Narayan, Misha B. Ahrens, Y. Albert Pan

**Affiliations:** ^1^Center for Neurobiology Research, Fralin Biomedical Research Institute at VTC, Virginia Tech, Roanoke, VA, United States; ^2^Janelia Research Campus, Howard Hughes Medical Institute, Ashburn, VA, United States; ^3^Department of Biomedical Sciences and Pathobiology, Virginia-Maryland College of Veterinary Medicine, Virginia Tech, Blacksburg, VA, United States; ^4^Department of Psychiatry and Behavioral Medicine, Virginia Tech Carilion School of Medicine, Roanoke, VA, United States

**Keywords:** transsynaptic, zebrafish, VSV (vesicular stomatitis virus), TRAS-M51R, brain mapping, viral tracing

## Abstract

The small size and translucency of larval zebrafish (*Danio rerio*) have made it a unique experimental system to investigate whole-brain neural circuit structure and function. Still, the connectivity patterns between most neuronal types remain mostly unknown. This gap in knowledge underscores the critical need for effective neural circuit mapping tools, especially ones that can integrate structural and functional analyses. To address this, we previously developed a vesicular stomatitis virus (VSV) based approach called Tracer with Restricted Anterograde Spread (TRAS). TRAS utilizes lentivirus to complement replication-incompetent VSV (VSVΔG) to allow restricted (monosynaptic) anterograde labeling from projection neurons to their target cells in the brain. Here, we report the second generation of TRAS (TRAS-M51R), which utilizes a mutant variant of VSVΔG [VSV(M51R)ΔG] with reduced cytotoxicity. Within the primary visual pathway, we found that TRAS-M51R significantly improved long-term viability of transsynaptic labeling (compared to TRAS) while maintaining anterograde spread activity. By using Cre-expressing VSV(M51R)ΔG, TRAS-M51R could selectively label excitatory (*vglut2a* positive) and inhibitory (*gad1b* positive) retinorecipient neurons. We further show that these labeled excitatory and inhibitory retinorecipient neurons retained neuronal excitability upon visual stimulation at 5–8 days post fertilization (2–5 days post-infection). Together, these findings show that TRAS-M51R is suitable for neural circuit studies that integrate structural connectivity, cell-type identity, and neurophysiology.

## Introduction

Deciphering the connectivity patterns of neurons within the brain, both structural and functional, is necessary to fully understand how the brain functions. Many neural circuit mapping approaches have been developed, including those that utilize viral vectors as the transsynaptic label. In particular, rabies virus, adeno-associated virus (AAV), and pseudorabies virus have been widely used in mammalian species for both anterograde (labeling postsynaptic outputs) and retrograde (labeling presynaptic inputs) neural circuit tracing. However, the use of viral vectors in zebrafish, an increasingly important model for neuroscience, has been much more limited, as many of the common viral vectors have either no infectivity (AAV) or reduced ability to spread (rabies) when applied to zebrafish (Zhu et al., 2009; Dohaku et al., 2019).

To address this limitation, we explored the use of vesicular stomatitis virus (VSV), which has been successfully used as polysynaptic and monosynaptic transneuronal tracers in mice (Beier et al., 2013a,b). Expanding from mice, we found that VSV can be used for polysynaptic anterograde or retrograde polysynaptic transsynaptic tracing in zebrafish (Mundell et al., 2015; Beier et al., 2016). However, the unrestricted spread of replication-competent VSV makes it difficult to disambiguate between monosynaptic and polysynaptic connections.

**Figure 1:**
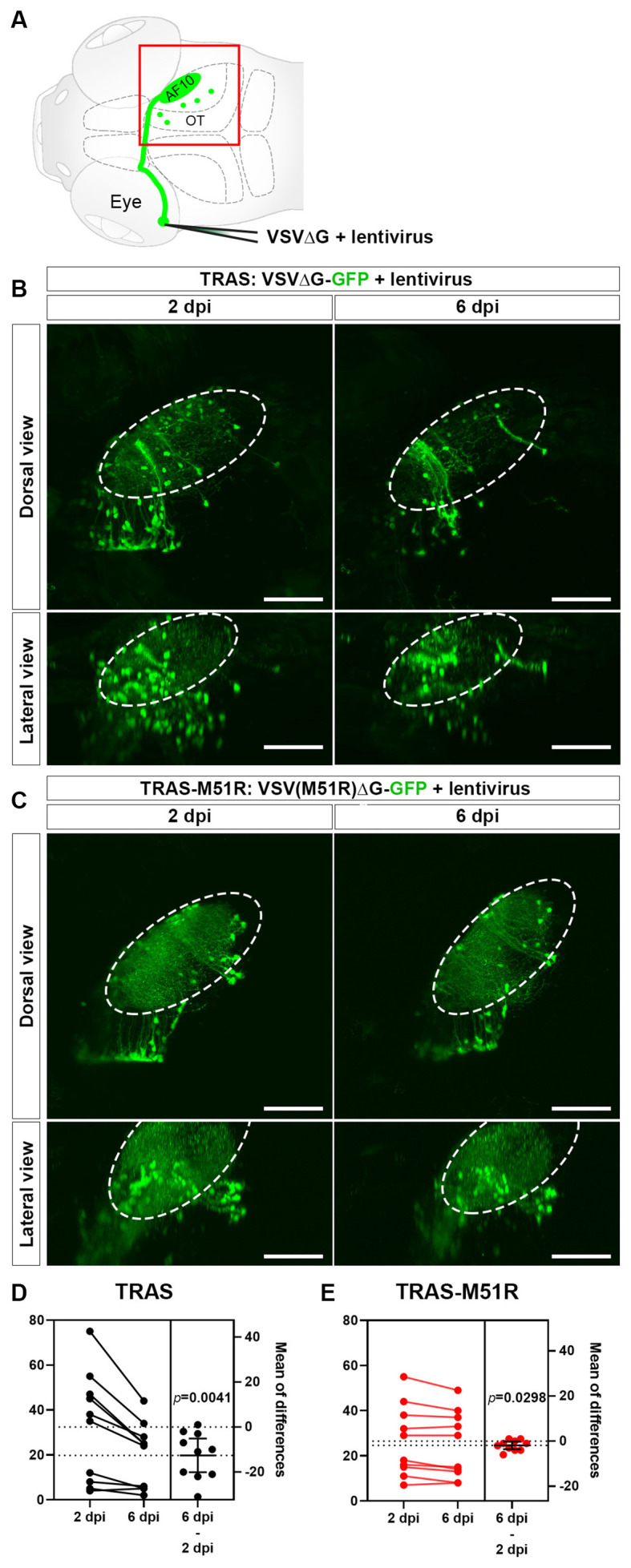
Comparison of VSVΔG and VSV(M51R)ΔG for TRAS labeling. **(A)** Schematic of TRAS labeling. G-deleted VSV (VSVΔG) and VSV-G pseudotyped lentivirus were co-injected into the temporal retina. This results in VSV infection and reporter gene expression in the RGCs in the retina and subsequent transsynaptic spread to retinorecipient cells in the contralateral brain (green circles). The red boxed area is shown in panels **(B,C)**. OT: optic tectum. AF10: RGC arborization field (AF) 10. **(B)** TRAS labeling with wild-type VSVΔG-GFP. The same animals were imaged at 2 and 6 days post-infection (dpi) [5 and 9 days post fertilization (dpf)], as indicated. Dorsal views are shown in the top row, and lateral views are shown in the bottom row. The number of VSVΔG infected cells (green cell bodies) was significantly diminished between 2 and 6 dpi. Labeling of RGC arbors within the optic tectum (fibers within the dashed outline) was also significantly reduced. **(C)** TRAS-M51R labeling with VSV(M51R)ΔG-GFP. The same animals were imaged at 2 and 6 dpi and shown in dorsal and lateral views, as indicated. The number of infected cells and the labeling of RGC arbors were similar between 2 and 6 dpi. **(D,E)** Estimation plots for changes in retinorecipient cell number between 2 and 6 dpi for TRAS and TRAS-M51R. The left part of the graph side shows the cell number for each fish at 2 and 6 dpi. Dashed lines indicate the means of each time point. The right side of the graph shows the effect size (mean of differences between 2 and 6 dpi). Error bars show the mean and 95% confidence interval (CI). The CI in **(D)** and **(E)** do not overlap with 0, indicating a significant decrease in cell number. *p* values from the two-tailed paired *t-*test are shown on graphs. Scale bars are 100 μm.

Recently, we developed Tracer with Restricted Anterograde Spread (TRAS) to address these limitations (Ma et al., 2020). Instead of replication-competent VSV, TRAS utilizes VSVΔG, which lacks the glycoprotein (*G*) gene and cannot spread on its own. We then complemented VSVΔG by providing external G-protein, in the form replication-incompetent lentivirus coated with VSV glycoprotein. By coinjection of VSVΔG and lentivirus, VSVΔG was able to spread to the direct synaptic partners of the initially infected neurons (starter cells). VSVΔG cannot spread further, as there is no longer lentivirus in the vicinity to facilitate spread. Using TRAS, we mapped and characterized the central target neurons of retinal ganglion cells (RGCs) and identified connectivity changes caused by genetic lesion of *dscaml1*, a neuronal cell adhesion molecule linked to human neuropsychiatric disorders (Iossifov et al., 2014; Karaca et al., 2015; Ma et al., 2020; Figure 1A).

To improve TRAS, we focused on reducing VSV’s significant neuropathic effects, which is one of the main limitations of TRAS and VSV-based neural circuit tracing approaches. VSV infection induces changes in the host cell’s physiology that favor the amplification of viral particles and inhibits the host cell’s antiviral responses. The inhibition of host-directed gene expression causes part of the cytopathic effects of VSV. This is achieved through blocking host cell translation, transcription, and nuclear export of mRNA (Ahmed and Lyles, 1998; Connor and Lyles, 2002). The VSV matrix (M) protein plays a significant role in these effects on host gene expression, which are separate from the M protein’s structural roles in the assembly and budding of nascent virions. As such, mutant variants of the M protein may potentially be utilized to develop VSV vectors that are less cytopathic but can still propagate sufficiently robustly to enable transneuronal spread.

The VSV *ts*O82 mutant was first identified as a temperature-sensitive mutant that can amplify in chick embryo fibroblasts only under permissive temperatures (Flamand, 1970). Molecular genetic studies then identified the causative mutation as a single methionine to arginine amino acid change at position 51 (M51R) of the VSV matrix (M) protein (Coulon et al., 1990). The M51R variant is fully functional in viral assembly, but is unable to inhibit host-direction mRNA synthesis (Kaptur et al., 1995), has reduced ability to inhibit translation (Connor and Lyles, 2002), and has reduced pathological nuclear export of hnRNPs (Pettit Kneller et al., 2009). These reduced cytopathic effects allow *ts*O82 to infect cultured cells chronically while minimizing cytotoxicity. In the context of neural circuit tracing, VSVΔG harboring the M51R mutation [VSV(M51R)ΔG] has been shown to spread transsynaptically in cultured mouse neurons while preserving the host cell’s ability to respond to synaptic activity (Beier et al., 2011). However, whether VSV(M51R) ΔG can be used as a neural circuit tracer *in vivo* remains undetermined.

In this study, we validated that VSV(M51R)ΔG can be used as an *in vivo* neural circuit tracer in larval zebrafish. Compared to the previous TRAS approach, TRAS with VSV(M51R)ΔG resulted in significant reductions in neuronal cytotoxicity while retaining robust spread from primary infected neurons (RGCs) to their monosynaptic targets. We name this new method TRAS-M51R. Furthermore, we incorporated the Cre-Lox combinatory gene expression system into TRAS-M51R, which enabled selective labeling of retinorecipient neuronal subtypes. Using the cell-type-specific expression of a genetically encoded calcium indicator, GCaMP6s, we show that retinorecipient neurons are viable post-infection for multiple days, and visual stimuli can evoke neuronal activity in retinorecipient neurons. Together, we demonstrate that this improved TRAS-M51R method is a robust technique to uncover novel structural and functional connectivity patterns in the vertebrate nervous system.

## Materials and Methods

### Zebrafish Husbandry

Zebrafish (all ages) were raised under a 14/10 light/dark cycle at 28.5°C. Developmental stages are as described by Kimmel et al. (1995). Embryos and larvae were raised in water containing 0.1% Methylene Blue hydrate (Sigma-Aldrich). Embryos were transferred to E3 buffer containing 0.003% 1-phenyl-2-thiourea (PTU; 207250250; Fisher Scientific) to prevent pigment formation at 24 h post-fertilization. For animals used for calcium imaging, animals were transferred back to media without PTU after virus injection [3 days post fertilization (dpf)]. Sex was not a relevant variable for this study, as laboratory zebrafish remain sexually undifferentiated in the stages being used (0–8 dpf; Maack and Segner, 2003; Wilson et al., 2014). All procedures were performed in accordance with Institutional Animal Care and Use Committee guidelines at Virginia Tech.

### Transgenic and Mutant Zebrafish Lines

The *vglut2a:LoxP-RFP-LoxP-GFP* [*TgBAC(slc17a6b:LoxP-DsRed-LoxP-GFP)^nns14^*], *gad1b:LoxP-RFP-LoxP-GFP* [*TgBAC(gad1b:LoxP-RFP-LoxP-GFP)^nns26^*], and *vglut2a:LoxP-RFP-LoxP-Gal4* [*TgBAC(slc17ab:LOXP-mCherry-LOXP-GAL4FF)^nns21^*] lines were provided by Dr. Shin-ichi Higashijima at the National Institute for Basic Biology (Koyama et al., 2011; Satou et al., 2013). The *UAS:GCaMP6s* [*Tg(5xUAS-hsp70l:GCaMP6s*] line was provided by Dr. Koichi Kawakami at the National Institute of Genetics (Muto et al., 2017). The *gad1b:LoxP-RFP-LoxP-Gal4* line was generated using the CRISPR-mediated knock-in method, as described by Kimura et al. (2014). The sgRNA for the *gad1b* locus is TGGAACTGCTCACCAGAAGG. Animals used for calcium imaging were in homozygous *nacre* (*mitfa*) mutant background (Lister et al., 1999).

### Virus Preparation

The pVSV(M51R)ΔG-GFP plasmid encoding the viral anti-genome was generously provided by Dr. Connie Cepko (Harvard Medical School/HHMI). To generate VSV(M51R)ΔG-Cre, the pVSV(M51R)ΔG-GFP plasmid was modified by replacing the GFP reporter gene with Cre-V5-mKate2. pVSV(M51R)ΔG-GFP and pVSV(M51R)ΔG-Cre plasmids were used to rescue virions *in vitro*, using a protocol modified from Witko et al. (2006). In brief, the plasmid encoding the VSV anti-genome was co-transfected with plasmids encoding VSV genes (pCMV-N, pCMV-P, pCMV-M, pCMV-G, and pCMV-L) and T7 RNA polymerase (pCMV-T7) into 293T cells in 6-well plates, using Lipofectamine 2,000 (Thermo Fisher Scientific). The plasmids encoding VSV genes and T7 RNA polymerase were gifted by Dr. Chris Parks (International AIDS Vaccine Initiative). The amount of plasmid used per well is shown in Supplementary Table 1. Following transfection, cells were heat-shocked at 43°C for 3 h and then incubated at 33°C for 4 days until GFP [for VSV(M51R)ΔG-GFP] or mKate2 [for VSV(M51R)ΔG-Cre]-positive foci appeared. The culture medium was collected for subsequent amplification and purification. We did not observe any mKate2 fluorescence after VSV(M51R)ΔG-Cre infection *in vivo*, despite robust Cre activity.

The rescued virus was further amplified on 293T cells transfected with pCI-VSVG (Addgene) plasmid and incubated at 32°C for 2 days. The medium was collected, and VSV was purified using ultracentrifugation as previously described (Ma et al., 2020). Typical viral titer was higher than 1 × 10^9^ focus forming units/ml (ffu/ml). Lentivirus stocks (lentivirus-SIN-CMV-eGFP or lentivirus-SIN-Ubi-iCre-mCherry) were purchased from the GT3 Core Facility of the Salk Institute. The titer range for lentivirus stock was 10^11^ to 10^12^ ffu/ml. All procedures for handling and preparation of viral vectors were approved by the Virginia Tech Institutional Biosafety Committee.

### Virus Injection

Viral injections were performed as previously described (Beier et al., 2016; Ma et al., 2020). Briefly, VSV and lentivirus stock was diluted to final working concentrations (described in the "Results" section) with DMEM (11995073; Fisher Scientific), with Fast Green dye (BP123-10; Fisher Scientific) as the injection marker. Glass capillaries (TW100F-4; World Precision Instruments) were pulled into injection needles with a pipette puller (P-97; Sutter Instruments), and the tips were trimmed to create a ~10 μm opening. The injection needle was mounted into a microelectrode holder connected to a pneumatic PicoPump (World Precision Instruments). For retina injection, 3 dpf larvae were anesthetized in Tricaine (0.013% w/v, AC118000500; Fisher Scientific) and mounted laterally inside the center chamber of a glass-bottom dish (P50G-1.5-14-F; MatTek) with 1.5% low melting-point agarose (BP1360; Fisher Scientific). 0.5 nl of virus solution was injected inside the temporal retina.

### Image Acquisition and Analysis

Images were acquired using a Nikon A1 confocal system with a CFI75 Apochromat LWD 25× water immersion objective. All images were acquired from live zebrafish larvae embedded in low-melting-point agarose, either anesthetized with Tricaine or un-anesthetized (for calcium imaging). Anesthetized animals were immobilized in low-melting-point agarose in glass-bottom dishes, with the dorsal side down (against the glass bottom). The dishes were then flipped over (dorsal side up) for imaging. Un-anesthetized animals were immobilized in low-melting-point agarose, dorsal side up, in Petri dishes containing medium, with agarose surrounding the eyes removed. Cell counting was done using the Cell Counter plugin (Plugins > Analyze > Cell Counter) in Fiji (Schindelin et al., 2012). Statistical analyses were done using the Prism 9 software (GraphPad). Spectral overlap between RFP and GFP channels in *vglut2a:LRL-GFP* and *gad1b:LRL-GFP* images were linearly unmixed in Fiji, using non-injected fish as references (Roossien and Cai, 2017).

### Visual Stimulation and Calcium Imaging

All visual stimulation during calcium imaging was controlled by Matlab (MathWorks) and Psychophysics Toolbox Version 3 (PTB-3). Some of the codes were modified *via* codes from http://www.peterscarfe.com/ptbtutorials.html, and projected through an LED projector (P3B 800-lumens ultra-short-throw; ASUS) onto a curved white arena. Projector display and laser onset are simultaneous. The first 60 (30 frames) are used as visual adaptation and not used for the analysis. The average calcium signal of the “Blank” period, which duration varies due to visual stimulus type, is used as the baseline average (F_0_), F_t_ starts after the adaptation period.

Confocal images were acquired at 512 × 512, 0.5 Hz, 305–320 frames. Structural images were acquired at 512 × 512, 2 μm z-step. Visual stimuli animals were presented with based on the area identifiable anatomically to detect functionality; some animals were presented with moving gratings, light flash, or both. Using the structural scans of each fish (5–6 dpf), the anatomical locations are then referenced to the ZBrain 3D brain atlas for identification and confirmation (Randlett et al., 2015). ROI marking and extraction of neuron fluctuations were achieved using Fiji Plot z-axis profile function (Image > Stacks > *Plot z*-*axis profile*). No noise filter was applied to measurements of calcium activities [(F_t_−F_0_)/F_0_].

## Results

### The M51R Variant of VSVΔG Exhibits Anterograde Transneuronal Spread and Reduced Cytotoxicity *In vivo*

The M protein of VSV is one of the principal contributors of cytotoxicity, causing cell rounding and inhibiting host cell-directed mRNA and protein synthesis. Engineered VSV harboring the methionine to arginine mutation in the M protein (M51R) has reduced inhibition of host-directed transcription and translation and may help preserve neuronal vitality during neural circuit mapping analyses. To test this, we first examined whether VSV(M51R)ΔG can be used in place of VSVΔG (with wild-type *M* gene) for TRAS-mediated monosynaptic labeling. We will refer to this new generation of VSV(M51R)ΔG-based TRAS as TRAS-M51R.

For TRAS-M51R, we co-injected GFP-expressing VSV(M51R)ΔG with VSV-G coated (pseudotyped) lentivirus into the zebrafish retina at 3 dpf. This resulted in robust labeling of retinorecipient cells along the optic tract at 2 days post-infection (dpi). TRAS with wild-type VSVΔG-GFP and lentivirus at the same titers resulted in similar labeling patterns (Figures 1B,C). One notable difference between TRAS and TRAS-M51R was in the RGC terminals in the optic tectum neuropil (arborization field 10, AF10; Burrill and Easter, 1994), where labeling is more robust in TRAS-M51R labeled animals. When the same animals were imaged at 6 dpi, the difference in optic tract labeling became more pronounced, and the RGC terminal arbors in AF10 was mostly absent in TRAS-labeled animals (Figures 1B,C). This result indicated that TRAS-M51R is less toxic to the RGCs. The preservation of retinal inputs with TRAS-M51R likely preserves the visual responsiveness of the targeted retinorecipient cells. We will explore this possibility in later sections.

To compared the cytotoxicity of TRAS and TRAS-M51R, we quantified the extent of cell loss within each animal between 2 and 6 dpi (Figures 1D,E). In the TRAS group, there was a substantial decrease in retinorecipient cell number (two-tailed paired *t-*test, *p* = 0.0041, mean difference = −12.60 ± 3.30 cells, *n* = 10 animals). In the TRAS-M51R group, there was also a significant reduction in cell number, but to a much smaller extent (two-tailed paired *t* test, *p* = 0.0298, mean difference = −1.90 ± 0.73 cells, *n* = 10 animals). These results show that the TRAS-M51R causes less cytotoxicity to starter cells (RGCs) and target (retinorecipient) cells.

### Cre-Dependent Labeling of Specific Retinorecipient Neuron Subtypes

In our previous study, we found that TRAS labeled three distinct populations of retinorecipient cells: excitatory neurons, inhibitory neurons, and a large population (50–60% of all TRAS-labeled cells) of putative non-neuronal cells (negative for the pan-neuronal transgene, *elavl3:GCaMP6f*; Ma et al., 2020). These three cell groups are distinguishable using a combination of transgenic labeling (*elavl3:GCaMP6f* transgene to label neurons) and immunohistochemistry (anti-GABA to label inhibitory cells). However, to further understand the structural and functional properties of neural circuits, it is imperative to restrict labeling to neurons, and ideally, to specific neuronal cell types. As VSV has its own transcriptional machinery that is not compatible with eukaryotic cell type-specific promoters, we explored options to use VSV to induce cell type-specific transcription by the host cell.

In rodent viral tracing studies, cell-type-specific labeling is often achieved by combining Cre-expressing viruses and transgenic animals carrying Cre-dependent reporter transgenes. Given that VSV(M51R) has low cytotoxicity and is permissive for host cell-directed gene expression, we tested whether Cre-expressing VSV(M51R)ΔG can be used to label specific retinorecipient neuron subtypes.

Cre-expressing VSV(M51R)ΔG [VSV(M51R)ΔG-Cre] was rescued *in vitro* from cDNA clones encoding the VSV anti-genome (Witko et al., 2006). Amplified and purified VSV(M51R)ΔG-Cre (1 × 10^9^ ffu/ml) was then coinjected with lentivirus (3 × 10^10^) into the left retina at 3 dpf. We used two transgenic reporter lines, *vglut2a:LoxP-RFP-LoxP-GFP* (*vlugt2a:LRL-GFP*, Koyama et al., 2011) and *gad1b:LoxP-RFP-LoxP-GFP* (*gad1b:LRL-GFP*, Satou et al., 2013), to label glutamatergic (excitatory) and gabaergic (inhibitory) neurons, respectively. RFP is expressed by default in both lines, and GFP is expressed when Cre excises the LoxP-RFP-LoxP sequence. GFP expression was assessed at 2 dpi. The ipsilateral (left) brain was used as the internal control, as retinal projections in zebrafish brain are strictly contralateral (Burrill and Easter, 1994).

**Figure 2:**
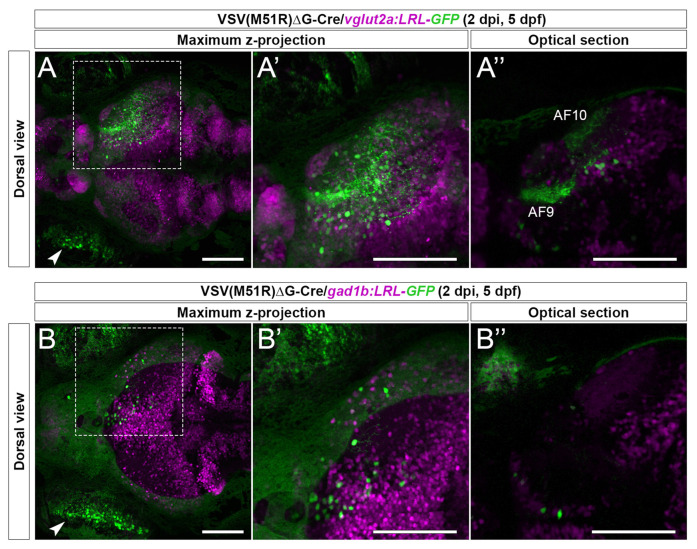
Cre-dependent TRAS-M51R labeling of specific retinorecipient neuron subtypes. **(A–A”)** Representative image of a 2 dpi (5 dpf) live *vglut2a:LRL-GFP* larvae injected with VSV(M51R)ΔG-Cre. Boxed area in **(A)** is shown in higher magnification in (**A’**; both show maximum z-projection). Panel **(A”)** shows a single optical section in the box area in **(A)**, at the level of AF9. GFP expression was seen in the injected eye (arrowhead in **A**), retinorecipient cells (round green cells), and the AFs (AF9 and AF10 shown in **A”**). **(B–B”)** Representative image of a 2 dpi (5 dpf) live *gad1b:LRL-GFP* larvae injected with VSV(M51R)ΔG-Cre. Boxed area in **(B)** is shown in higher magnification in **(B’)** (both show maximum z-projection). Panel **(B”)** shows a single optical section in the box area in **(B)**, at the level of AF9. GFP expression was seen in the injected eye (arrowhead in **B**) and retinorecipient cells. No AF labeling was seen. Green fluorescence in the uninjected eye was from the autofluorescence of retinal pigment epithelial cells. Scale bars are 100 μm.

As expected, in *vglut2a:LRL-GFP* animals (*n* = 5) we observed robust GFP expression in the RGC arborization fields (AFs) and cell bodies within the retinorecipient regions (Figures 2A–A”). In contrast, in *gad1b:LRL-GFP* animals (*n* = 2), the AFs were not labeled, and only retinorecipient cells express GFP (Figures 2B–B”). The lack of GFP expression in the AFs of *gad1b:LRL-GFP* line is consistent with RGCs being glutamatergic. There was no GFP expression in the ipsilateral brain in each transgenic line. Together, these results show that TRAS-M51R with Cre permits selective labeling of excitatory neurons or inhibitory neurons in live animals.

### Live Imaging of Calcium Dynamics in Retinorecipient Subtypes After TRAS-M51R Labeling

The integration of structural and functional connectivity properties is a critical component in neural circuit analyses. We have previously shown that TRAS labeling was suited to map structural connectivity, but whether it is compatible with physiological analyses has not been examined due to rapid cell loss (Figure 1D). Given the improved cellular viability with TRAS-M51R, we tested whether it is feasible to integrate TRAS-M51R with functional calcium imaging to assess neural activity.

We first examined the excitatory (glutamatergic) retinorecipient neurons. VSV(M51R)ΔG-Cre (3 × 10^8^ ffu/ml) and lentivirus (1 × 10^11^ ffu/ml) were coinjected into the *vglut2a:LoxP-RFP-LoxP-Gal4;UAS:GCaMP6s* double transgenic fish (*vglut2a:LRL-Gal4;UAS:GCaMP6s*). In this transgenic line, excitatory neurons express RFP by default and GCaMP6s in the presence of virus-transduced Cre expression. At 2–5 dpi (5–8 dpf), live animals were presorted for GCaMP6s (green) fluorescence, mounted in low-melting-point agarose, and imaged under a confocal microscope. Two types of visual stimuli were presented to the animals during imaging: moving gratings and whole-field light flashes (see “Materials and Methods” section). Overall, 71.4% of all imaged animals (10 of 14, 5–8 dpf) had GCaMP6s-positive glutamatergic neurons that responded to visual stimulations. All animals (14 of 14, 5–8 dpf) had visual-driven activity within the AFs of the RGC axons.

**Figure 3:**
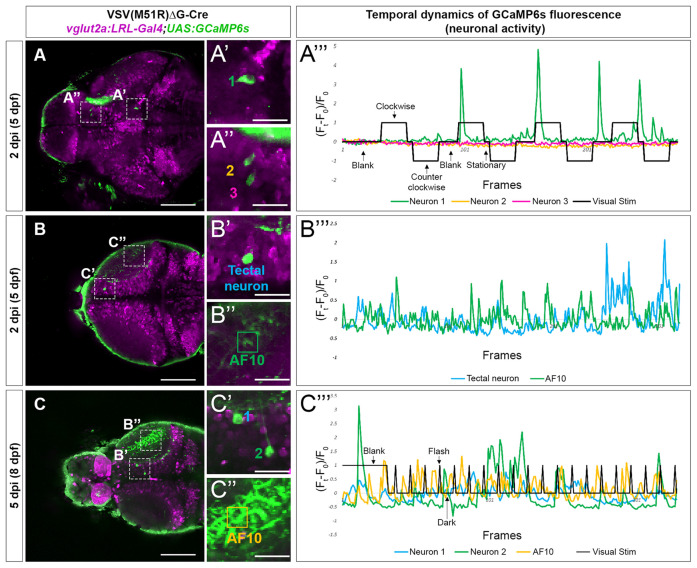
Temporal dynamic of calcium activities in multiple conditions captured from neurons infected with VSV(M51R)ΔG-Cre in *vglut2a:LRL-Gal4;UAS:GCaMP6s* fish. All images shown are confocal optical sections. **(A–A”’)** Neurons (**A’**—neuron in Tegmentum—NucMLF and **A”**—Dorsal Thalamus neurons) expressing GCaMP6s detected in one XY plain of the brain in a 2 dpi (5 dpf). The evoked transient calcium activity traces (F_t_ − F_0_/F_0_) during whole field display of moving gratings is shown in **(A”’)**. **(B–B”’)** Anterior tectal neuron **(B’)** and AF10 **(B”)**, expressing GCaMP6s detected in one XY plain of the brain in a 2 dpi (5 dpf) fish. The spontaneous transient calcium activity traces during uniform illumination (blank) is shown in **(C”’)**. **(C–C”’)** Tectal neurons **(C’)** and AF10 **(C”)**, expressing GCaMP6s detected in one XY plain of the brain in a 5 dpi (8 dpf) fish. The evoked transient calcium activity traces during whole-field display of 18 s dark/2 s bright flashes is shown in **(C”’)**. Scale bars in** (A–C)** are 100 μm. Scale bars in **(A’,A”,B’,B”,C’,C”)** are 25 μm.

As the typical visual responsivities of many retinorecipient brain regions are known (Baier and Wullimann, 2021), we identified the location of TRAS-M51R labeled retinorecipient neurons using ZBrain, a digital 3D zebrafish brain atlas (Randlett et al., 2015). The brain region names (capitalized) are based on the annotations in Zbrain 1.0^1^. In one 5 dpf (2 dpi) animal, we identified a TRAS-M51R labeled glutamatergic neuron in the mesencephalon, within the Tegmentum—NucMLF (nucleus of the medial longitudinal fascicle; Neuron 1, Figures 3A–A”’). When this animal was presented with a moving grating stimulus (which triggers optokinetic eye movements), we found that Neuron 1’s calcium flux peaked at the onset of grating motion (the second and third trial of “Clockwise” motion) and the end of the grating motion (“Blank” and “Stationary” before and after the fourth trial of “Clockwise” motion). This activity suggests that Neuron 1 is transiently tuned to changes in whole-field motion, which is consistent with NucMLF’s involvement in sensorimotor control and eye movements (Walter and Shaikh, 2014). In contrast, two Dorsal Thalamus neurons in the same animal (Neuron 2 and Neuron 3) had weak responses to the same visual stimulus (Figures 3A–A”’). This finding is consistent with previous findings that the dorsal thalamus does not respond strongly to moving grating stimuli (Baier and Wullimann, 2021). Some animals had neurons with apparent spontaneous calcium activity (3 out of 5 at 5 dpf). In the example shown in Figures 3B–B”’, the animal was presented with a blank white blank screen. The neuron in the Tectum—Stratum Periventriculare region and the Tectum—Neuropil (AF10) exhibited spontaneous calcium flux under this uniform luminance. Robust calcium flux was observed up to 8 dpf (5 dpi), as shown in Figures 3C–C”’. The animal was presented with a blank white screen (60 s), followed by 2 s light flashes. Calcium flux was seen both during the blank and flash periods, but did not occur in a strict event-related manner.

**Figure 4:**
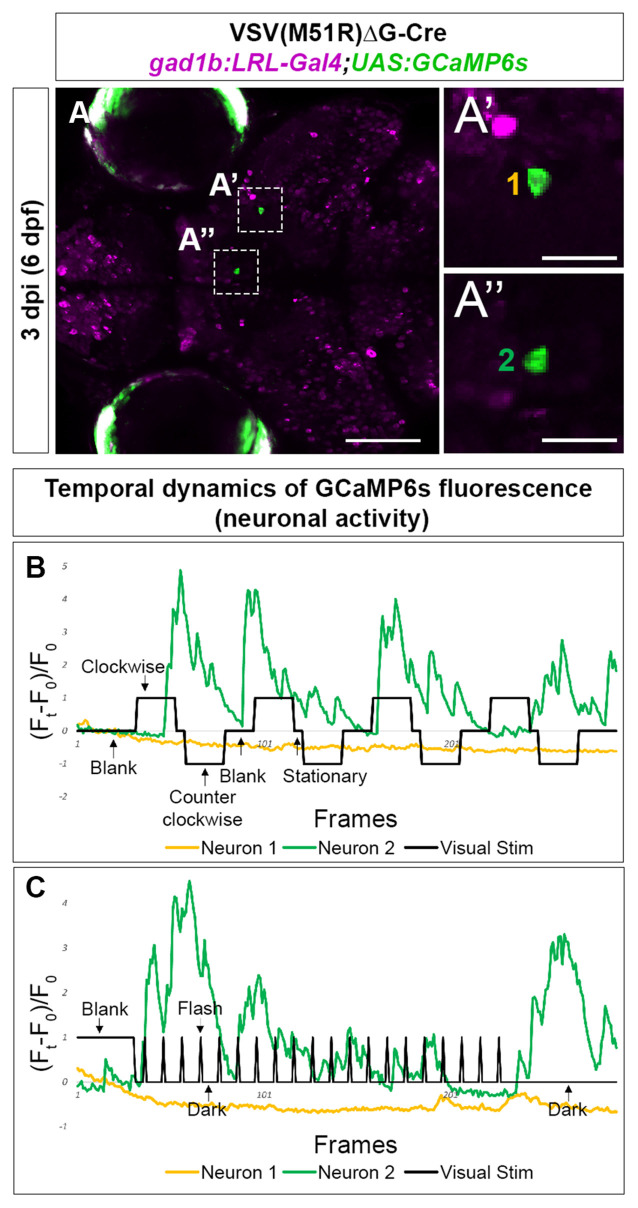
Temporal dynamic of calcium activities in multiple conditions captured from the neurons infected with VSV(M51R)ΔG-Cre in *gad1b:LRL-Gal4;UAS:GCaMP6s* fish. All images shown are confocal optical sections. **(A–A”)** Neurons (**A’**—Mesencephalon neuron and **A”**—Dorsal Thalamus neuron) expressing GCaMP6s detected in one XY plain of the brain in a 3 dpi (6 dpf) fish. **(B)** The evoked transient calcium activity traces (F_t_ − F_0_/F_0_) during the whole-field display of moving gratings. **(C)** The evoked transient calcium activity traces during whole field display of 18-s dark/2-s bright flashes. Scale bar in **(A)** is 100 μm. Scale bars in** (A’,A”)** are 25 μm.

Next, we examined the inhibitory (gabaergic) retinorecipient neurons using the same labeling methods and viral titers but a different transgenic line, *gad1b:LoxP-RFP-LoxP-Gal4;UAS:GCaMP6s* (*gad1b:LRL::GCaMP6*). At 3–4 dpi (6–7 dpf), we found that all imaged animals (5 of 5) had TRAS-labeled gabaergic neurons that responded to visual stimuli. We also observed visual-driven activity in labeled neurites within the AFs. As RGCs are not labeled in this transgenic line, this AF-associated activity is likely derived from TRAS-labeled retinorecipient cells. In the animal shown in Figure 4, both moving gratings (Figure 4B) and light flashes (Figure 4C) were presented. Neuron 1, located in the mesencephalon, did not show calcium flux associated with the stimulus. In contrast, Neuron 2, located in the Dorsal Thalamus, exhibited calcium flux when presented with both types of visual stimuli. Calcium activity of Neuron 2 appears to be amplified during luminance reduction, at the transition from whole-field illumination (blank) to dark (Figure 4C). This is consistent with the finding that the zebrafish visual thalamus responds to drops in luminance (Heap et al., 2018).

Together, we show that TRAS-M51R is compatible with functional imaging under physiological stimuli. The visual stimuli-associated responses are partially synchronized with the stimulations given, reflecting certain features of those stimuli and are generally consistent with previous zebrafish calcium imaging studies. Notably, the integration of the Cre-dependent expression system opens the door to the targeted labeling and recording of specific retinorecipient cell types, as we have shown here with the *vglut2a* and *gad1b* expressing glutamatergic and gabaergic neurons, respectively.

## Discussion

In this study, we report an improved viral-based anterograde transsynaptic neural circuit tracing method, TRAS-M51R, which significantly improved long-term neuronal labeling compared to the previously described TRAS method. The apparent reduction in cytotoxicity afforded by the M51R variant allows it to induce transgene expression in the host cell in a Cre-dependent manner. When combined with cell type-specific reporter lines, we could induce GFP or GCaMP6s expression selectively in retinorecipient excitatory and inhibitory neurons. These improvements address one of the main limitations of TRAS, which is the high level of cytotoxicity in both the starter cells (RGCs) and target cells (retinorecipient cells). The selective labeling of excitatory and inhibitory neurons with VSV(M51R)ΔG-Cre also eliminates the extensive labeling of abundant non-neuronal cells present with TRAS.

Reducing the cytotoxicity of viral vectors is a prerequisite to permit functional studies after neural circuit labeling. Commonly used transsynaptic viruses such as rabies virus, pseudorabies virus, and VSV all have notable cytotoxic effects that prevent the normal function of neurons. Even relatively benign viruses such as AAV can induce cell death and alter neuronal activity and neurogenesis (Johnston et al., 2021). Notably, a recent study has shown that a different VSV variant (R7A mutation in the VSV-N protein) may have reduced toxicity and enhanced anterograde spread in mice (Lin et al., 2020). Combining the R7A and M51R mutations may further improve VSV as a tool for combined structural and functional neural circuit analysis. Another critical area of future development is the specificity of the initial infection. As the VSV glycoprotein can bind to most cell types, TRAS-M51R is best suited to target projection neurons that innervate more distant brain regions. An orthogonal viral glycoprotein-membrane receptor system (e.g., EnvA-TvA) will be needed to distinguish between initial infection and local spread.

As with any viral neural circuit tracing method, prospective users should consider that the efficiency and specificity of transsynaptic spread depend on numerous factors, including injection site, synaptic strength, viral titer, and host immune response (Rogers and Beier, 2021). Our previous work found that TRAS predominantly labels monosynaptic afferent target areas (Ma et al., 2020). However, multisynaptic spread can occasionally occur at high viral titer, likely due to the recycling of VSV-G in the postsynaptic cell. Thus far, we have not observed any polysynaptic spread with TRAS-M51R, but it remains possible if higher viral titers or immunocompromised animals were used. It will be prudent to confirm novel connectivity findings with alternative approaches such as electron microscopy or neurophysiology.

In recent years, there has been rapid progress in developing other viral vectors for zebrafish neurobiology, particularly the use of the rabies virus for retrograde transsynaptic labeling (Dohaku et al., 2019; Satou et al., 2021). However, only VSV has been successfully applied for transsynaptic labeling in the larval zebrafish, a critical stage for behavioral and functional imaging studies. As such, the TRAS-M51R method fills a critical need for viral neural circuit mapping tools in the larval zebrafish.

## Data Availability Statement

The raw data supporting the conclusions of this article will be made available by the authors, without undue reservation.

## Ethics Statement

The animal study was reviewed and approved by The Institutional Animal Care and Use Committee at Virginia Tech.

## Author Contributions

SK, MM, and YP conceived the study, designed the experiments, performed the experiments, and analyzed the data. SN and MA generated transgenic animals used for Calcium imaging analysis. YP wrote the manuscript with extensive input from SK and MM. All authors contributed to the article and approved the submitted version.

## Conflict of Interest

The authors declare that the research was conducted in the absence of any commercial or financial relationships that could be construed as a potential conflict of interest.

## Publisher’s Note

All claims expressed in this article are solely those of the authors and do not necessarily represent those of their affiliated organizations, or those of the publisher, the editors and the reviewers. Any product that may be evaluated in this article, or claim that may be made by its manufacturer, is not guaranteed or endorsed by the publisher.

## Footnotes

^1^ https://zebrafishexplorer.zib.de/home/

## References

[B1] Ahmed M. Lyles D. S. (1998). Effect of vesicular stomatitis virus matrix protein on transcription directed by host RNA polymerases I, II and III. J. Virol. 72, 8413–8419. 10.1128/JVI.72.10.8413-8419.1998 9733895 PMC110232

[B2] Baier H. Wullimann M. F. (2021). Anatomy and function of retinorecipient arborization fields in zebrafish. J. Comp. Neurol. 529, 3454–3476. 10.1002/cne.25204 34180059

[B3] Beier K. T. Borghuis B. G. El-Danaf R. N. Huberman A. D. Demb J. B. Cepko C. L. (2013a). Transsynaptic tracing with vesicular stomatitis virus reveals novel retinal circuitry. J. Neurosci. 33, 35–51. 10.1523/JNEUROSCI.0245-12.2013 23283320 PMC3711516

[B6] Beier K. T. Saunders A. B. Oldenburg I. A. Sabatini B. L. Cepko C. L. (2013b). Vesicular stomatitis virus with the rabies virus glycoprotein directs retrograde transsynaptic transport among neurons *in vivo*. Front. Neural Circuits 7:11. 10.3389/fncir.2013.00011 23403489 PMC3566411

[B4] Beier K. T. Mundell N. A. Pan Y. A. Cepko C. L. (2016). Anterograde or retrograde transsynaptic circuit tracing in vertebrates with vesicular stomatitis virus vectors. Curr. Protoc. Neurosci. 74, 1.26.1–1.26.27. 10.1002/0471142301.ns0126s74 26729030 PMC4776322

[B5] Beier K. T. Saunders A. Oldenburg I. A. Miyamichi K. Akhtar N. Luo L.. (2011). Anterograde or retrograde transsynaptic labeling of CNS neurons with vesicular stomatitis virus vectors. Proc. Natl. Acad. Sci. U S A 108, 15414–15419. 10.1073/pnas.1110854108 21825165 PMC3174680

[B7] Burrill J. D. Easter S. S. Jr. (1994). Development of the retinofugal projections in the embryonic and larval zebrafish (Brachydanio rerio). J. Comp. Neurol. 346, 583–600. 10.1002/cne.903460410 7983245

[B8] Connor J. H. Lyles D. S. (2002). Vesicular stomatitis virus infection alters the eIF4F translation initiation complex and causes dephosphorylation of the eIF4E binding protein 4E-BP1. J. Virol. 76, 10177–10187. 10.1128/jvi.76.20.10177-10187.2002 12239292 PMC136556

[B9] Coulon P. Deutsch V. Lafay F. Martinet-Edelist C. Wyers F. Herman R. C.. (1990). Genetic evidence for multiple functions of the matrix protein of vesicular stomatitis virus. J. Gen. Virol. 71, 991–996. 10.1099/0022-1317-71-4-991 2157808

[B10] Dohaku R. Yamaguchi M. Yamamoto N. Shimizu T. Osakada F. Hibi M. (2019). Tracing of afferent connections in the zebrafish cerebellum using recombinant rabies virus. Front. Neural Circuits 13:30. 10.3389/fncir.2019.00030 31068795 PMC6491863

[B11] Flamand A. (1970). Genetic study of vesicular stomatitis virus: classification of spontaneous thermosensitive mutants into complementation groups. J. Gen. Virol. 8, 187–195. 10.1099/0022-1317-8-3-187 4321566

[B13] Heap L. A. L. Vanwalleghem G. Thompson A. W. Favre-Bulle I. A. Scott E. K. (2018). Luminance changes drive directional startle through a thalamic pathway. Neuron 99, 293–301.e4. 10.1016/j.neuron.2018.06.013 29983325

[B14] Iossifov I. Sanders S. J. Ronemus M. Krumm N. Levy D. Stessman H. A.. (2014). The contribution of de novo coding mutations to autism spectrum disorder. Nature 515, 216–221. 10.1038/nature13908 25363768 PMC4313871

[B15] Johnston S. Parylak S. L. Kim S. Mac N. Lim C. Gallina I.. (2021). AAV ablates neurogenesis in the adult murine hippocampus. eLife 10:e59291. 10.7554/eLife.59291 34259630 PMC8331179

[B16] Kaptur P. E. McKenzie M. O. Wertz G. W. Lyles D. S. (1995). Assembly functions of vesicular stomatitis virus matrix protein are not disrupted by mutations at major sites of phosphorylation. Virology 206, 894–903. 10.1006/viro.1995.1012 7856102

[B17] Karaca E. Harel T. Pehlivan D. Jhangiani S. N. Gambin T. Akdemir Z. C.. (2015). Genes that affect brain structure and function identified by rare variant analyses of mendelian neurologic disease. Neuron 88, 499–513. 10.1016/j.neuron.2015.09.048 26539891 PMC4824012

[B18] Kimmel C. B. Ballard W. W. Kimmel S. R. Ullmann B. Schilling T. F. (1995). Stages of embryonic development of the zebrafish. Dev. Dyn. 203, 253–310. 10.1002/aja.1002030302 8589427

[B19] Kimura Y. Hisano Y. Kawahara A. Higashijima S. (2014). Efficient generation of knock-in transgenic zebrafish carrying reporter/driver genes by CRISPR/Cas9-mediated genome engineering. Sci. Rep. 4:6545. 10.1038/srep06545 25293390 PMC4189020

[B20] Koyama M. Kinkhabwala A. Satou C. Higashijima S. Fetcho J. (2011). Mapping a sensory-motor network onto a structural and functional ground plan in the hindbrain. Proc. Natl. Acad. Sci. U S A 108, 1170–1175. 10.1073/pnas.1012189108 21199937 PMC3024692

[B21] Lin K. Zhong X. Ying M. Li L. Tao S. Zhu X.. (2020). A mutant vesicular stomatitis virus with reduced cytotoxicity and enhanced anterograde trans-synaptic efficiency. Mol. Brain 13:45. 10.1186/s13041-020-00588-3 32197632 PMC7085170

[B22] Lister J. A. Robertson C. P. Lepage T. Johnson S. L. Raible D. W. (1999). Nacre encodes a zebrafish microphthalmia-related protein that regulates neural-crest-derived pigment cell fate. Development 126, 3757–3767. 10433906 10.1242/dev.126.17.3757

[B23] Ma M. Kler S. Pan Y. A. (2020). Structural neural connectivity analysis in zebrafish with restricted anterograde transneuronal viral labeling and quantitative brain mapping. Front. Neural Circuits 13:85. 10.3389/fncir.2019.00085 32038180 PMC6989443

[B24] Maack G. Segner H. (2003). Morphological development of the gonads in zebrafish. J. Fish Biol. 62, 895–906. 10.1046/j.1095-8649.2003.00074.x

[B25] Mundell N. A. Beier K. T. Pan Y. A. Lapan S. W. Goz Ayturk D. Berezovskii V. K.. (2015). Vesicular stomatitis virus enables gene transfer and transsynaptic tracing in a wide range of organisms. J. Comp. Neurol. 523, 1639–1663. 10.1002/cne.23761 25688551 PMC4458151

[B26] Muto A. Lal P. Ailani D. Abe G. Itoh M. Kawakami K. (2017). Activation of the hypothalamic feeding centre upon visual prey detection. Nat. Commun. 8:15029. 10.1038/ncomms15029 28425439 PMC5411483

[B27] Pettit Kneller E. L. Connor J. H. Lyles D. S. (2009). hnRNPs relocalize to the cytoplasm following infection with vesicular stomatitis virus. J. Virol. 83, 770–780. 10.1128/JVI.01279-08 19004954 PMC2612367

[B28] Randlett O. Wee C. L. Naumann E. A. Nnaemeka O. Schoppik D. Fitzgerald J. E.. (2015). Whole-brain activity mapping onto a zebrafish brain atlas. Nat. Methods 12, 1039–1046. 10.1038/nmeth.3581 26778924 PMC4710481

[B29] Rogers A. Beier K. T. (2021). Can transsynaptic viral strategies be used to reveal functional aspects of neural circuitry? J. Neurosci. Methods 348:109005. 10.1016/j.jneumeth.2020.109005 33227339 PMC7856199

[B30] Roossien D. H. Cai D. (2017). “Imaging neural architecture in brainbow samples,” in Site-Specific Recombinases: Methods and Protocols, ed Eroshenko N. (New York: Springer), 211–228. 10.1007/978-1-4939-7169-5_14 28815503

[B31] Satou C. Kimura Y. Hirata H. Suster M. L. Kawakami K. Higashijima S. (2013). Transgenic tools to characterize neuronal properties of discrete populations of zebrafish neurons. Development 140, 3927–3931. 10.1242/dev.099531 23946442

[B32] Satou C. Neve R. L. Oyibo H. K. Bouldoires E. A. Mori T. Higashijima S.. (2021). A viral toolbox for conditional and transneuronal gene expression in zebrafish. BioRxiv [Preprint]. 10.1101/2021.03.25.436574 PMC9307271 35866706

[B33] Schindelin J. Arganda-Carreras I. Frise E. Kaynig V. Longair M. Pietzsch T.. (2012). Fiji: an open-source platform for biological-image analysis. Nat. Methods 9, 676–682. 10.1038/nmeth.2019 22743772 PMC3855844

[B34] Walter B. L. Shaikh A. G. (2014). “Midbrain,” in Encyclopedia of the Neurological Sciences, eds Aminoff M. J. Daroff R. B. (Oxford: Academic Press), 28–33.

[B35] Wilson C. A. High S. K. McCluskey B. M. Amores A. Yan Y. L. Titus T. A.. (2014). Wild sex in zebrafish: loss of the natural sex determinant in domesticated strains. Genetics 198, 1291–1308. 10.1534/genetics.114.169284 25233988 PMC4224167

[B36] Witko S. E. Kotash C. S. Nowak R. M. Johnson J. E. Boutilier L. A. Melville K. J.. (2006). An efficient helper-virus-free method for rescue of recombinant paramyxoviruses and rhadoviruses from a cell line suitable for vaccine development. J. Virol. Methods 135, 91–101. 10.1016/j.jviromet.2006.02.006 16569439

[B37] Zhu P. Narita Y. Bundschuh S. T. Fajardo O. Scharer Y. P. Chattopadhyaya B.. (2009). Optogenetic dissection of neuronal circuits in zebrafish using viral gene transfer and the tet system. Front. Neural Circuits 3:21. 10.3389/neuro.04.021.2009 20126518 PMC2805431

