# Supplementary material for: Cre-Dependent Anterograde Transsynaptic Labeling and Functional Imaging in Zebrafish Using VSV With Reduced Cytotoxicity
Source: Front Neuroanat. 2021 Oct 6;15:758350. doi: 10.3389/fnana.2021.758350 (PMC8549678; doi:10.3389/fnana.2021.758350)
Supplement: Supplementary file 1 [file Table_1.DOCX]

| Plasmid | DNA per well (ng) |
| --- | --- |
| pT7 | 1400 |
| pVSV(M51R)ΔG-Cre | 250 |
| pN | 200 |
| pP | 100 |
| pL | 30 |
| pM | 30 |
| pG | 400 |

**Supplementary Table 1. Amount of plasmid DNA used for the rescue of VSV(M51R)ΔG-Cre**
